# Supplementary material for: Are carbon emissions trading and green financial instruments synergistic? -Comprehensive quantitative research based on content analysis
Source: PLoS One. 2024 Mar 7;19(3):e0298601. doi: 10.1371/journal.pone.0298601 (PMC10919601; doi:10.1371/journal.pone.0298601)
Supplement: S1 Data — (ZIP) [file pone.0298601.s001.zip › Data/地级市Z_F工作报告.zip!/║┌┴·╜¡/╥┴┤║╩╨/└┤╫╘╣1⁄2╓┌║┼í╛┬φ┐╦╩2╛▌═°í┐_2008.docx]

各位代表：

现在，我代表市政府，向大会作工作报告，请予审议，并请市政协各位委员和其他列席人员提出意见。

一、2007年工作回顾

2007年是市政府换届后的第一年，在省委、省政府和市委的正确领导下，市政府团结带领全市人民，从伊春实际出发，全面落实科学发展观，坚持“生态立市”、“工业兴市”发展战略，深化国有林权制度改革，加大森林资源保护力度，积极对上争取转型政策，全力打造发展环境，真心实意解决民生问题，求真务实，开拓创新，圆满地完成了市十二届人大一次会议确定的主要目标和工作任务，开创了经济社会发展的新局面。全市生产总值完成153.8亿元，同比增长11.5％。全口径财政总收入完成8亿元，同比增长30.5%，其中地方财政收入完成4.5亿元，同比增长35.1%，是财政形势最好的时期。固定资产投资完成50.1亿元，同比增长30.5％。

持之以恒保护森林资源，生态建设取得重大成果。“生态立市”战略深入人心，“绿色伊春”的影响进一步扩大。严管资源的各项举措得到有效落实，木材生产和资源林政管理得到全面加强。木材售价创历史最高水平，销售收入比去年同期增收1亿元，严管林效果进一步显现。森林抚育4.7万公顷，更新造林1.5万公顷，面积合格率达99.5%，成活率达94.5%。加大投入，积极治理，有效控制了舞毒蛾对森林资源的侵害。乌伊岭湿地晋升为国家级自然保护区。金山屯、带岭连续实现半个多世纪无森林火灾的业绩，走在了全国森林防火工作前列。在伊春历史上首次倡导并成功举办了2007森林生态保护（伊春）国际论坛，29个国家的代表一致通过的《森林生态保护伊春宣言》，受到了国内外的广泛关注和支持。在伊春国有林区全面停止森林主伐、创建天保工程示范区的建议得到了国家有关部门的认同。在停伐红松的基础上，发行了中国第一张以“关注伊春，保护红松”为主题的《红松龙卡》，设立了红松保护基金，成立了伊春保护红松联合会，开展了认领红松行动。在全国率先启动博士后创新团队“碳汇经济”项目，将森林的固碳减排和林区风电等清洁发展机制项目作为可持续发展的支柱产业。这些一开先河的重要举措，对森林生态的保护和发展、促进生态平衡、走可持续发展之路都将起到至关重要的作用。我市先后被世界自然基金会定为中国五个低碳试点城市之一，在联合国第七届全球论坛上被评为“世界十佳和谐城市”。

林权制度改革试点取得阶段性成果，各项改革稳步推进。顺利完成了8万公顷国有林权制度改革试点任务。经过一年多的实践，改革取得了显著的阶段性成效，显示出了强大的生机与活力。推动了国有林区的体制机制创新，初步建立起责权利相统一的森林资源管理新机制；激发了林业职工培育和保护森林资源的积极性，促进了森林资源的科学经营和可持续发展；极大地释放了林业生产力，拓宽了林业职工就业和致富渠道。承包职工自费造林完成3313公顷。所有承包林地未发生一起林政案件和森林火情火警。林下经济发展方兴未艾。涌现了联户承包、家庭林场、股份合作林场等新型经济组织。国家林业局主持召开的国有林权制度改革试点研讨会高度评价了改革试点工作，为推进国有林权制度改革向纵深发展提供了重要理论和政策支持。国有企业改革不断深化，中小企业改制基本完成，对已改制企业进行了“回头看”，提高了改制效果。西钢集团和西林铅锌矿改制后企业主辅分离和职工有关待遇得到了妥善处理。晨明水泥厂破产清算前期准备工作完成，带岭造纸厂政策性破产进入法定程序。事业单位改革试点稳步推进，全面推行了新进人员聘用和公开招考制度。行政审批制度改革不断深入，所有审批事项及收费项目全部纳入行政服务中心办理。金融、非税收入收缴制度、财政国库集中支付制度等各项改革都取得了新成效。

产业结构不断优化，经济发展后劲进一步增强。农业结构调整不断深化，粮食产量达到57.9万吨，同比增长9.8%。工业调整改造取得了新成效，规模以上工业企业实现增加值26.4亿元，同比增长13.2%，全部工业对地区生产总值增长的贡献率达到38.2%。西钢120吨转炉、朗顺强化地板和朗乡、桃山风力发电等重点项目相继投产，加快了新型工业化进程，促进了经济转型。金融保险、商贸流通、交通运输、社区服务等第三产业活力增强，特别是森林生态旅游业开始火爆，旅游接待人数达300万人，实现收入12亿元，同比增长27.2%。五大接续替代产业增加值占GDP的比重达到35.1%。所有制结构调整加快，全市中小企业发展到6.6万户。从事自营经济的职工家庭14.3万户，户均收入达1.7万元。非国有经济实现增加值112.5亿元，占GDP比重的73.2%。

“三农”工作扎实推进，新农村建设取得新进展。各项支农惠农政策得到有效落实。进一步扩大了新农村建设试点的覆盖面和基础薄弱试点单位比例。加大了对新农村建设试点的投入，省、市财政和帮建单位对两级试点共投入资金1240万元。粮食种植面积达到263万亩，优质品种推广应用达90%以上，农业机械化程度达到81.5%，绿色食品原料种植基地达到170万亩，无公害农产品、绿色食品、有机食品认证达到101个，铁力市30万亩国家级绿色水稻标准化生产基地通过验收。生态畜牧业发展态势良好，产值占农业总产值的40%以上。农村基础设施建设力度加大，完成农村通畅通达公路建设968公里，新建饮水工程57处，新打灌溉抗旱井382眼，修复水毁灌溉工程16处，沼气、太阳能等清洁能源得到广泛推广，农村D级危房进行了全面改造。全市农村合作医疗参合率达到93％。

招商引资成果不断扩大，对外开放迈出新步伐。成功举办了第七届森林生态旅游节、2007·黑龙江伊春·原生态食用菌发展论坛暨首届山特产品交易会、第三届中国（伊春）冬季摄影节等节事活动。借助中央电视台“朝闻天下”金牌栏目，使“中国林都、红松故乡、黑龙江伊春”名传天下。与三亚市缔结为友好城市，签署了旅游合作协议。我市被评为“2007年中国十佳避暑旅游城市”和“2007中国特色魅力城市200强”。组织企业参加了“哈洽会”、“广交会”等一系列经贸洽谈会，在深圳、杭州、骊水、天津、三亚等地举办了旅游经贸推介会，提高了招商引资的实效性，加强了对外交往。全年国内引资36.6亿元、实际利用外资1176万美元、实现外贸进出口总额1.3亿美元。对俄经贸合作不断升级，嘉荫口岸实现过货8万吨，增加了成品油、汽车等新的过货品种，向俄输出劳务2540人，在俄采伐木材80万立方米，出入境人员7533人次。

基础设施不断完善，城乡面貌变化显著。“四区连片”步伐加快。市中心城供热改造项目在国家开发银行立项，资金已陆续到位。一级供热管网和路灯分别延伸至翠峦区、友好区。完成了主街路面、人行道板和临街建筑物外墙的改造。盛世荣府、星河湾二期等一批住宅小区相继建成。新建经济适用住房25万平方米，改造危旧房屋8万平方米，落实廉租住房保障资金200余万元，完成漏雨楼房平改坡1.3万平方米，铺设供水管线54千米，新增集中供热面积30万平方米、公共绿地面积17.8公顷，移植大树进城10万株，绿化亮化美化工程建设提高了城市品位和档次。我市被确定为全省棚户区改造唯一试点城市，棚户区改造项目获得省国家开发银行批准。伊春林都机场项目完成了动迁和场地整平，机场路已开工建设。兴安湖水库、中心城污水处理厂等城市重点工程进展顺利。小城镇建设力度加大，各县（市）区（局）城镇面貌发生显著变化。公路交通网逐步完善，建设完成了嘉荫县保兴山支线工程，加抚公路逊嘉界至稻田段工程量完成97%，鹤岗至哈尔滨高速公路伊春段、绥嘉公路嘉荫至保兴段项目正在开展前期工作，完成了6个客运运输场站建设项目，城市聚集和辐射功能进一步加强。

节能减排措施有力，资源节约和环境保护取得实效。加大对重点耗能企业改造力度，能源利用效率进一步提高。6个项目列入国家《松花江流域水污染防治规划》。发动社会力量开展了保护母亲河--汤旺河行动。加速淘汰落后产能、工艺和设备进程，取缔落后设备5个，改造锅炉34台，取缔锅炉46台，拆除烟囱77根。否决了不符合国家产业政策、选址不合理的项目16个。对70家环境违法企业限期整改，对属于“十五小”的3家企业予以取缔，对11家已有污染防治设施企业实行了严格监管。全市工业烟尘排放达标率、工业废水排放达标率均达到90％，工业固体废物综合利用率达到60%。全市城市环境空气质量达到国家二级以上标准天数为360天，达标率为98.6%，城市集中饮用水源地水质达标率为100%。

人民生活明显改善，各项社会事业全面发展。集中财力重点加大了民生支出比重，民生状况得到较大改善。就业保障、教育科技、医疗卫生、文化体育支出比例分别增长35%以上，是全市历史上投入最大的一年。优先解决工资收入问题，职工工资收入大幅度增加，先后三次调资，年调资总额达5.7亿元。调整了市财政供养范围内的行政事业单位在职及离退休人员工资，月人均增资383元，高于省补助标准83元；增调了森工企业、政企合一区（局）机关事业单位在职及离退休人员工资，在岗职工月人均增资375元，退休人员月人均增资100元，将南四局及森工院墙企业也纳入了本次调资范围，比省补助人数多42286人，全市林业在岗职工月人均工资达到736元；增加了市本级在职及离退休人员工资津贴补贴，在职人员月人均增资300元，比省补助标准高186元，离退休人员分别按在职人员的90%和85%予以补贴。三次调资市财政年多增加支出1.26亿元。城镇居民人均可支配收入实现8010元，农民人均纯收入实现4675元，比2006年分别增长8%和13%。城乡居民储蓄存款余额达到155.5亿元，同比增长2.6%。就业和再就业工作取得较大成绩，城镇新增就业再就业4.4万人，零就业家庭实现动态为零，城镇居民登记失业率控制在4%。社会保障体系进一步完善，各类社会保险参保率稳步提高，森工系统职工医疗保险实现市级行业统筹。城市最低生活保障面已达到11.7%，13万多城镇困难居民纳入保障范围，1.2万人纳入农村低保，人均年补助400元以上。对五保供养对象提高了供养标准。3956户特困职工在第四轮扶贫解困工程中实现脱贫，脱贫率达到44%。对6.8万名森工企业混岗人员进行了一次性安置。2万多名政企合一区（局）地方大集体职工纳入森工企业混岗人员安置政策。科技创新能力进一步提高，组织实施市以上新产品开发等重点科技项目42项，取得科技成果20项，获得专利14项，新增民营科技企业5户。教育事业全面加强，办学条件明显改善，新建、维修、改造校舍15万平方米，教育教学管理得到加强，教育质量大幅度提高，除伊春区外，义务教育阶段学杂费全部免除。文化事业繁荣发展，信息、通讯、广播电视网络建设不断完善，“基层文化年”活动丰富多彩，优秀新闻作品和文艺精品不断涌现。话剧《青山常在》应邀在中央党校演出，在全国“群星奖”大赛和“蒲公英”选拔赛当中我市多个作品获奖。医疗卫生事业全面发展，市、区两级惠民医院和大病救助资金普遍建立，林场卫生所全面恢复，农村卫生网点全面加强，社区卫生服务机构转型建设全面启动，小病不出场所、村屯目标基本实现，群众就医难问题基本得到解决。“兴安健康行”活动全面开展，10万人享受到了免费体检政策。计划生育工作继续稳定在低生育水平。《妇女儿童发展规划》顺利实施。群众体育广泛开展，竞技体育水平不断提高，速滑项目水平跻身于全国前列，成功举办了市第八届运动会。精神文明建设水平不断提高，双拥共建继续推进，第四次被评为“全国双拥模范城”。民主法制建设不断加强，普法教育和依法治理成效显著。平安伊春创建不断深入，社会治安形势保持平稳。安全生产责任制得到落实，产品质量和食品安全专项整治行动取得初步成效。民族宗教、外事侨务、人防、边防等项工作都取得了新成绩。

各位代表，同志们，以上成绩的取得，是省委、省政府和市委正确领导的结果，是人大、政协积极支持的结果，是全市人民戮力同心，团结奋斗的结果，是民主党派、社会各界人士广泛参与的结果。在此，我代表市政府向辛勤工作、勇于奉献的全市人民致以崇高的敬意，向驻伊部队、武警官兵、中省直单位和所有关心、支持伊春发展的各界人士表示衷心的感谢！

各位代表，同志们，在看到所取得成绩的同时，我们也应该清醒地看到，还有许多深层次矛盾、多年积累的问题尚未得到根本解决。主要是：思想解放不够，改革开放意识不强；少数领导干部靠消耗森林资源搞建设的思想仍然存在，破坏森林资源的现象和法人超采行为仍有发生；烧柴改革和工业减排步履蹒跚，环境保护和节能降耗任务依然艰巨；经济发展方式转变缓慢，接续替代产业支撑力不强；企业自主创新能力较弱，高科技项目凤毛麟角；财政收支矛盾依然突出，投融资渠道不够畅通；就业形势不容乐观，社会保障能力亟待增强；职工工资近年来虽有较大幅度增长，但与省内经济发达地区相比仍然存在差距；政府个别部门职能转变还不到位，对基层和企业检查过多，经济发展环境仍需改善。对于这些问题，我们将高度重视，采取措施，认真加以解决。

二、2008年工作总体部署

2008年工作的总体要求是：以党的十七大精神为指导，认真落实中央经济工作会议和省委、市委十届四次全会的部署，坚持用科学发展观统领经济社会发展全局，深入实施“生态立市”、“工业兴市”战略。积极保护发展森林资源，加强资源节约和环境保护，建设生态文明。加大林权制度改革和经济转型力度，推进改革开放和自主创新，转变经济发展方式。加快社会发展，着力改善民生，构建林区和谐社会。实现经济、政治、文化、社会建设协调发展，全面建设社会主义新林区。

2008年全市经济社会发展的主要预期目标是：在优化结构、提高效益的基础上，生产总值增长10%；地方财政收入比上年预算增长10.1%；规模以上工业增加值增长13%；全社会固定资产投资增长25%；社会消费品零售总额增长13%；国内引资、实际利用外资、外贸进出口分别比上年实际增长20%、10%、10%；城镇居民人均可支配收入增长7.1%；农民人均纯收入增长6%；万元GDP综合能耗下降4.5%，城镇登记失业率控制在4%以内。

实现上述目标，重点抓好以下九个方面的工作。

(一)以保护和培育森林资源为重点，推进生态功能区建设

继续加大严管资源力度。深入实施“天保工程”，坚定不移地执行“严管林”方针，强化采伐限额管理，杜绝法人超采，严惩偷拉私运、毁林盗伐等涉林违法行为，严肃追究超采单位法人代表及相关人员的行政或刑事责任。加强热爱森林保护生态宣传教育，扩大《森林生态保护伊春宣言》国内国际影响。全面落实保护红松的各项举措，加强与俄罗斯、韩国、朝鲜等国家的沟通与合作，举办“保护红松国际研讨会”，促进保护红松国际联盟建立，使保护红松行动成为全社会的共同行动，永远保持伊春祖国林都、红松故乡的美誉。在政策支持到位的情况下，在全市范围内全面停止天然林主伐，加快我市森林资源根本性恢复，保障国家生态安全。主动转变木材经销观念，利用林木经销信息网络资源，搜集发布外埠木材经销信息，搭建木材流通交易平台，变木材流出为木材输入，增加木材供给量。整合林产工业企业，提高木材精深加工水平，节约林木资源，提高经济效益。强化林政监督检查，完善“漏斗式”管理模式。继续推行木材联网竞价销售，促进木材增收。加强林地管理，从严控制征用占用林地，坚决制止农林交错地区蚕食林地行为，积极恢复废弃林地森林植被，防止林地逆转。加强森林资源的资产化管理。加强北药资源保护、培育和开发，严厉打击地道野生药材乱采滥挖、掠夺性采收等破坏行为。严厉打击滥捕乱猎不法行为，切实保护好林蛙等野生动物资源。在林农交错地区继续推广乌伊岭、红星林业局吸收农民参与管护经营的经验。加强森林防火工作，强化重点火险区的治理，提高病虫鼠害科技防治水平，确保森林资源安全。

切实搞好森林培育。制定红松资源建设规划，建立完善红松繁育基地，增加红松育苗数量，加大红松培育比例，加快红松更新造林步伐。调整更新方式，增加人工促进更新和人天混更新比重。优化林种树种结构，增加阔叶珍贵树种的造林比重，大面积营造混交林、阔叶林、速生丰产原料林和经济林，完成森工和地方更新造林8.36万亩，红松、混交林造林比例分别达到25%和30%。严格执行森林抚育技术规程，完成森林抚育114万亩，提高森林质量和林地生产力，加速森林的正向演替进程。

加强自然保护区建设。强化自然保护区规范化建设，做好生态功能区区划和现有自然保护区晋档升级工作。优化整合自然保护区体系结构，新建市级自然保护区1个，申报晋升国家级自然保护区1个。

（二）以林权制度改革为重点，推进各项改革向纵深开展

全面深化林权制度改革。国有林权制度改革试点已进入“总结经验、完善政策、深化试点”的新阶段。按照国务院领导批示精神和国家林业局的总体部署，认真研究落实伊春国有林权制度改革试点研讨会的各项要求，加大工作力度，推进国有林权制度改革向纵深发展。认真总结试点经验，进一步完善林改后的经营措施，着力引导承包户科学经营，提高林地生产力。进一步完善承包经营服务体系，全方位为承包经营职工提供优质服务。积极对上争取林木权属证书发放、林地抵押贷款、林木资产保险、产权交易等林权改革配套政策。适时启动活立木交易市场，规范林地使用权、林木所有权流转，实现国有林区盘活活立木资产的历史性突破。力争扩大试点面积，放大改革效应。做好林农交错地带的林权制度改革试点工作，保障农林交错地带森林资源的安全。

进一步深化国有企业改革。重点抓好晨明水泥厂、带岭造纸厂破产清算工作，尽快实现破产终结。积极争取天保工程政策，对13户森工企业实施政策性破产。对规模以上尚未改制的国有企业，以实现投资主体多元化为重点，进一步推进产权制度改革。对公益性单位结合事业单位改革，在国有控股或参股的基础上，吸引非国有资本，逐步实行放开经营。对优势特色产业中具有竞争优势的企业，采取招大联强，国有控股、参股的形式进行集团化改造，加快建立现代企业制度步伐。继续推进国有大中型企业主辅分离和辅业改制工作，尽快解决西钢集团、西铅改制遗留问题。加大林业局和林场所改革力度，转变职能，加快转型，实现由木材生产型向多种经营型、生产管理型向经营服务型转变。稳妥做好粮食流通体制改革。

继续推进事业单位改革。按照全省统一部署，加快推进事业单位分类改革和不同类型事业单位的岗位设置，大力推行事业单位新进人员面向社会进行公开招聘，实现事业单位用人的规范化、制度化。分类推进事业单位人事制度的配套改革，建立与聘用制和岗位管理制度相适应的各项考核制度。

大力发展非公有制经济。把加快非公有制经济发展作为全市结构调整和经济发展的重点，进一步落实促进非公有制经济发展的政策措施。努力整合社会资源，健全和完善中小企业服务体系，发挥翠东、带岭省级中小企业创业基地引领作用，深入开展“创办小企业、开发新岗位、以创业促就业”活动。扶持非公有制骨干企业，打造中小企业产业集群，提升非公有制经济规模、发展层次和整体素质。继续加大职工自营经济扶持力度，进一步在发展领域、整体规模、质量效益上实现扩张。

（三）以加快经济转型为重点，推进经济发展方式转变

紧紧抓住国务院出台《关于促进资源型城市可持续发展的若干意见》的有利机遇，认真研究相关政策，争取将我市纳入国务院首批资源枯竭城市名单。以林业棚户区改造为突破口，加大经济转型政策争取力度，加快转型步伐。

加快新型工业化进程。深入贯彻省委《加快新型工业化进程的决定》，抓好“全市新型工业发展规划”编制工作，加快生态型工业体系建设，提高资源精深加工能力和水平。深入实施大项目带动战略，加快发展五大接续替代产业，增强经济整体实力。抓好浩水二线、西林8万吨铅冶炼、西钢1080吨高炉、铁力局鹿鸣钼矿和新青老白山、金山屯老白山、带岭东山风力发电等投资亿元以上的重点项目，尽快落实开工建设。加快南岔广源、庆丰水电站和兴安新能源光伏电池、铁力市九河泉米业水稻加工、兴安红酒业、葵花药业、嘉荫高档复合地板、朗乡多层板等一批重点续建项目实施进度，使其尽快竣工投产。加快企业信息化进程，以信息化带动工业化。开发上下游产品，延伸上下游产业链，推进优势产业集群化、专业化、社会化。

推动企业自主创新。积极谋划一批创新项目，围绕冶金、制药、绿色能源、新材料等产业为重点，以大中型企业为主体，加大研发投入，推进企业技术中心建设，推动产学研联合，加快科技创新和信息技术应用，促进企业研发一批科技含量高、附加值大、市场竞争能力强的拥有自主知识产权的新技术和新产品，推动工业企业技术进步和优化升级。重点抓好兴安新能源太阳能供热系统开发、伊春药业多利欣等一批新产品研发项目，培育一批具有核心技术的产品，增强企业竞争力。

大力发展循环经济。制定并实施《伊春市循环经济发展规划》，完善发展循环经济各项推进机制。在重点耗能行业、企业中全面开展清洁生产和节能降耗工作，加快农村、林场所、城镇烧柴改革步伐，积极推广沼气、太阳能、生物质半气化多用炉技术等新型节能生活方式，实现资源投入的减量化、各种废弃物资源化和再利用。启动朗乡林业局林业循环经济试点工作，加快构建“四区三链”循环经济体系建设，形成低投入、低消耗、低污染、高效益的经济发展方式，努力实现清洁生产。

抓好节能减排工作。建立完善科学的污染减排指标、节能审计监测和考核体系。着力抓好重点行业、企业和重点工程的节能减排工作。坚决淘汰高耗能、高污染企业的落后产能、工艺装置和技术设备，鼓励企业利用先进技术和先进设备进行节能改造。严把新污染产生关，坚决杜绝违反国家限制、明令淘汰的落后工艺、设备以及能耗高、污染严重的项目落户伊春。积极发展碳汇经济项目，加快林业低碳城市建设步伐。

（四）以发展农村经济为重点，推进社会主义新农村、新林区建设

加快发展现代农业。以增加农民收入为出发点和落脚点，认真落实支农惠农政策。以发展农业产业化、集约化经营为切入点，积极推广现代农业科技，促进农业增产增收。加大“小兴安岭大森林”和“林都北药”品牌宣传力度，大力发展特色种养与加工企业。进一步扩大优质粮食种植面积，稳步发展地道北药和青贮饲料等高效的经济作物。加快无公害农产品、绿色和有机食品基地建设和认定步伐。大力发展黑木耳、猴头等原生态食用菌生产。培育壮大龙头企业，加快发展农产品精深加工和森工多种经营，加大市场营销力度，做大做强特色农业。积极扶持以奶牛、生猪为重点的常规养殖，鼓励发展特种养殖，不断提高规模化、标准化水平。

加强农村和林场所基础设施建设。加大投入力度，繁荣发展农村和林场所各项事业。继续实施乡镇集中供水、供电、通讯和通畅通达工程，解决农村和林场所饮用水、用电、行路难、就医难等问题，进一步改善农村和林场所生产生活条件。以病险水库、沿河堤坝除险加固和节水灌溉为重点，搞好农田水利建设，增加节水灌溉面积，提高农业综合保障能力。抢抓新农村建设的各种机遇，争取更多的政策支持。巩固和发展新农村建设试点成果，加大帮建工作力度，落实帮扶措施，发挥农民在新农村建设中的主体作用，高标准高质量地完成三年新农村建设试点任务。

大力发展劳务经济。把劳务经济作为农村和林场所新兴产业来抓，加强对农民和职工的技能培训，提高素质，促进灵活就业。积极开辟外埠劳务市场，加强劳务服务体系建设，引导农民组建各类协会，提高务工组织化程度。

（五）以生态旅游业为重点，推进第三产业加快发展

重点发展生态旅游业。出台《伊春市加快旅游业发展的决定》，解决旅游业发展中的重大问题。对全市各重点旅游区的开发进行统一规划，继续加大景区景点资源整合与建设力度。全面启动小兴安岭风景观光道建设，突出抓好旅游综合服务区建设，加快旅游景区精品建设，着力打造具有代表性的地质景观、森林景观和度假景区。整合地质遗迹资源，开展申报小兴安岭世界地质公园工作。加大旅游市场宣传力度，进一步开拓“珠三角”、“长三角”和境外高端客源市场，努力打造中国旅游休闲度假热线和“北有伊春、南有三亚”的旅游联盟品牌，切实形成山海呼应、南北互动的旅游格局。办好第八届中国黑龙江伊春森林生态旅游节、第四届中国（伊春）冬季摄影节等节事活动。培植发展旅游龙头企业，开发特色旅游商品。加强旅游人才培训，健全旅游服务体系。加快旅游交通、住宿等配套设施建设，提高旅游接待能力。加快旅游业由“接待游”向“产业游”转变，提升旅游支柱产业地位。

积极发展现代服务业。发挥旅游业的带动作用，加快发展金融、保险、信息、咨询、仓储、培训等成长性强的现代服务业和生产性服务业。大力推行连锁经营、特许经营、多式联运等新型商业模式，推动传统服务业管理信息化、服务标准化。继续实施“万村千乡”市场工程，积极引导和鼓励流通企业在农村和林场所建立连锁店、超市和“农家店”，扩大消费需求。支持物业管理、家政服务等社区服务业发展，引导居民建设家庭旅馆，进一步推动休闲旅游等热点消费，拓宽消费领域。

（六）以招商引资为重点，推进对外开放工作提档升级

积极开展招商引资。进一步改革完善招商引资激励机制，发挥企业招商引资的主体作用，以企招商、以商招商、以项目招商，提高招商引资质量。广泛挖掘可招商引资的项目和资源，充实招商引资项目库，统筹规划，分级落实。对符合产业政策、发展前景好的重点企业、重点项目进行认真梳理，在引进大企业、高科技项目和智力人才方面实现新的突破。发挥好伊春工业区的集聚效应，做好招商引资新建项目落户工业区工作。组织企业积极参加各类经贸洽谈活动，加大对外宣传推介力度。

扩大进出口规模。充分发挥我市自身产业产品结构特点和地缘区位优势，大力实施品牌战略，积极拓展外贸合作领域和空间，扩大出口产品的种类和规模，加大税收支持力度，落实退税政策，促进对外贸易协调稳步增长。

扩大对外经贸合作。以对俄经贸为重点，整合全市的资源，加大对俄能源、原材料的投资、合资和劳务输出力度，加快对俄农副产品基地、森林采伐基地的建设。积极开展与俄政府间、民间、建筑、旅游、科技、文化等领域的交流合作，推动我市对俄交流合作向全方位、多层次、宽领域发展。努力加强对美、日、韩和港、澳、台等国家和地区的合作，力争取得实质性进展。加强与周边地区和发达省份的合作，扩大与“长三角”、“珠三角”等经济区的协作。搞好与国外友好城市的合作和交流。

（七）以财源建设为重点，推进财税收入稳步增长

加强财源建设。充分发挥公共财政的作用，完善政策激励机制，推进县(市)区财源建设。充分发挥财政资金的引导作用，通过担保、贴息、奖励等方式，引导社会资金、金融资产、风险投资及其它资金支持循环经济、节能减排等重点项目建设。完善部门预决算制度、政府投资评审机制及财政风险预警机制。优化政府采购品种结构，加大工程项目的政府采购力度。

强化税收征管工作。严格执行税收法律法规和政策，完善税收管理制度，依法加强税收征管及重点行业的税务稽查，及时清缴欠税，严厉打击偷税行为，努力扩大财政收入总量。

优化支出结构。从严从紧控制一般性支出，重点保障教育、科技、农业、民生等项支出法定增长，提高社保、下岗再就业支出比重，合理安排公益性基础设施建设和环境保护等支出。加强非税收入管理，落实收支两条线规定，严肃财经纪律，强化地方财政和森工企业财务管理的审计监督和绩效评价。

大力改善金融环境。引导金融机构创新服务方式，提高服务质量，加大对地方经济的支持力度。加强政府、企业、公民诚信建设，建立健全银行、政府、企业三方协调沟通机制，破解信贷需求难题。进一步探索建立中小企业信用担保体系，引导企业通过市场资本运营，扩大融资渠道，逐步提高直接融资比重。严厉打击各种逃废金融债务的不法行为，全面创建金融安全区。

(八)以改善民生为重点，推进林区和谐社会建设

把促进社会发展和解决民生问题摆在更加突出的位置，继续集中财力、物力、人力，着力解决人民群众最关心、最直接、最现实的问题，让人民群众从改革发展中得到更多实惠。

加强就业再就业工作，完善社会保障体系。积极贯彻实施《劳动合同法》、《就业促进法》。认真落实就业再就业优惠政策，完善公共就业服务体系，建好社区就业服务平台，千方百计扩大就业岗位。加强职业技能培训，搞好就业再就业实习基地建设，提高就业本领。强化各级政府依法促进就业职能，帮助“零就业”家庭解决就业问题，统筹做好停伐后职工就业、城镇新增劳动力就业、农村富余劳动力转移就业、下岗失业人员再就业工作，加强对高校毕业生、退役军人的就业指导和服务。进一步扩大社会保险覆盖面,完善养老、工伤、失业保险制度，不断提高养老金、失业救济金、工伤人员待遇水平，逐步建立健全覆盖城乡全体居民的医疗保险体系，启动实施生育保险。切实加强社保资金的监督管理，规范运作，确保专款专用。加强劳动监察，落实最低工资制度。积极对上争取国家补助资金，努力解决历史遗留的拖欠职工工资问题。加大引导力度，促进城乡居民财产性收入稳步增长。继续开展“一帮一”扶贫解困工程，健全社会救助体系，广泛开展多层次、多种类、多形式的居家养老和集中养老服务，改善孤老集中供养条件。大力发展慈善事业，深入开展专项和经常性捐赠活动。落实城乡低保制度，提高补助标准，继续做到动态管理下的应保尽保。

提高城乡规划和建设管理水平，着力改善人居环境。加大生态园林城市依法建设力度，抓好中心城总体规划、控制性详细规划及村镇规划的修编、编制工作，搞好城市景观及建筑色彩的研究和设计工作，科学指导合理调控城乡协调发展。提升城镇绿化水平，按照“高中矮、针阔彩”的标准，由造林绿化向造景美化转变，提高景观效果和观赏价值。加快伊春林都机场、中心城污水处理厂、兴安湖水库、中心城垃圾处理厂等重大工程建设，继续完善供热、给排水体系，增强城市整体功能。全面实施廉租住房保障制度，完善和规范经济适用房制度，大力推进棚户区改造和旧小区综合整治，改善林区中低收入家庭和困难对象的住房条件。坚持经营城市理念，活化城市公共资源，做好城市各类公共资产的招标拍卖、冠名等工作。加强城市管理，抓好城管工作由主干道向次干道、居民小区延伸，绿化、美化由城市向农村延伸工作。加大城市环境综合整治力度，完善环境监测预警和监管体系，积极进行水、大气、土壤、噪声等污染防治，全力打造最佳人居环境。

大力发展社会事业，促进社会协调进步。均衡城乡教育资源，优化学校布局，促进教育公平。全部免除城镇义务教育阶段学生学杂费，全部免除农村义务教育阶段学生的书本费。继续加大教育投入,努力改善办学条件,全面优化教育发展环境。普及高中阶段教育，提高基础教育质量。大力发展职业教育，以伊春职业学院为龙头，加强高、中等职业院校建设。积极推进科技创新，建设创新型城市。加快创新型人才、高技能人才和各类紧缺人才的培养和引进，促进人才共享和柔性流动。大力开展科学普及和创新意识培养，加快技术成果转化和先进技术推广，实施省市级科技项目30项。加强文化建设，促进文化产业发展。深入开展社区群众文化活动，组织力量创作一批具有小兴安岭特色的文艺精品，开展第六届“大森林之声”系列文化活动，打造绿色伊春生态文化品牌。加强未成年人思想道德建设，大力实施“四自五爱”工程，争创“全国文明城市”，推动全市精神文明再上新台阶。深入落实《关于加快卫生事业改革与发展的决定》，建立完善基本医疗卫生制度，加强疾病防控、妇幼保健等技术服务机构建设，理顺各级卫生行政部门职能，加强各级医院管理，改进医德医风，规范药品价格，重点解决群众看病贵的问题，努力提高医疗卫生服务水平。大力发展体育事业，着力改善社区、广场等公共场所体育设施，开展全民健身活动，提高竞技体育水平。做好人口和计划生育工作，稳定低生育水平。积极发展老龄、妇女儿童、残疾人事业。认真抓好城市社区换届选举工作。加强农村社区建设，完善农村民主管理制度。发挥民间组织作用，提高社会服务功能。

强化公共安全，维护社会稳定。严格落实安全生产责任制，加大安全执法力度，强化对食品、药品、学校、公共聚集场所、非煤矿山、危险化学品、交通运输、烟花爆竹和消防安全等重点领域的专项整治，坚决遏制重特大安全事故发生。深化“平安伊春”建设，加强社会治安综合治理，建立完善覆盖城乡主要道路、重点部位的监控网络，遏制重特大刑事案件发生。坚持依法治访，探索信访工作新机制，做好重大社情民意的政策解释工作，解决好群众信访的突出矛盾和问题。完善各类应急预案，妥善处置突发性公共安全事件。认真贯彻落实各项优抚安置政策，保持和发展“全国双拥模范城”的荣誉。全面做好民族、宗教、外侨、档案、市志等各项工作。

（九）以提高工作效能为重点，推进政府自身改革和建设

进一步转变政府职能。各级政府及政府各部门要把主要精力放在为各类市场主体服务和创造良好发展环境上。深化行政审批制度改革，进一步减少和规范行政审批事项，坚决杜绝各种变相审批行为。改进公共服务方式，实行阳光办公，一条龙服务，着力把市行政服务中心建设成为政府为民、利民、便民的重要平台。加快“中国·伊春”政府门户网站建设步伐，进一步拓宽政务公开和为民服务渠道。

坚持依法行政。自觉接受人大及其常委会的监督、质询，主动向人大报告工作；自觉接受政协的民主监督，虚心听取对政府工作的意见和建议。继续深入贯彻《行政许可法》，规范行政执法行为，明确行政执法权限，完善执法程序，提高执法水平，努力建设法制政府。

加强廉政建设。坚持标本兼治、综合治理、惩防并举、注重预防的方针，坚持不懈地同各种腐败行为作坚决斗争。继续抓好领导干部的教育、监督和廉洁自律，继续抓好木材生产、加工、销售领域以及破坏森林资源、破坏生态环境等违法违纪案件查处，继续抓好并纠正损害群众利益的不正之风，着力解决人民群众最关心的价格和服务收费等热点难点问题。加强行政效能监察，建设务实廉洁高效政府。

各位代表，同志们，伊春的发展任重道远，人民的期望催人奋进。让我们高举中国特色社会主义伟大旗帜，以邓小平理论、“三个代表”重要思想和科学发展观为指导，在市委的正确领导下，继续解放思想，抢抓机遇，改革创新，为促进全市经济社会又好又快发展，建设富裕文明和谐的社会主义新林区而努力奋斗！
